# The Milk in the Cocoanut

**Published:** 1895-03

**Authors:** 


THE MILK IN THE COCOA-NUT.—For several weeks past, Cincinnati and New York papers have contained much stuff on the subject of consumption, written by two certain notorious parties who claim to have a "chemical cure" for that disease. The citizens of El Paso, Texas,—a favorite and well known resort for consumptives,—issued a protest against large numbers of indigent persons flocking to their town too far advanced in consumption to hope for climatic or any other kind of benefit, even if they were able to provide themselves with all the requisites of living. The protest was justifiable, from every standpoint, and was really in the interest of humanity, those poor creatures going to El Paso under the delusion that a few months residence in that climate will cure them. They become a burden to the city, and necessarily suffer.

About the same time, in Cincinnati, two hospital surgeons, very properly, separated the consumptives from others, and put them in a ward especially provided for them. It was a coincidence, we learn, that this ward had been at one time used for small-pox cases.

These Cincinnati "philanthropists" who have a chemical cure to sell, and are therefore purely philanthropic,—entirely disinterested,—thereupon rushed into columns of print, threatening to prosecute with all their money, power, influence, ability (and, we may add, gall), anybody who would dare to send a poor consumptive to a small-pox pest house, or to interfere with his going to El Paso, or elsewhere,--hades, if he wished, after his own fashion. (Oh, mighty men of valor! "Shoot *any*body's sheep that comes out in the middle of the road to bite me.")

We wrote Health Officer Yandell, at El Paso, inquiring what it was all about, any way; whence this tempest in a teapot; and here is his reply:

EL PASO, TEXAS, February 24, 1895.

*Editor Texas Medical Journal:*

DEAR DOCTOR:—Yours of the 21st inst. received yesterday, too late for answer that day. The El Paso Board of Health appointed a committee to prepare resolutions setting forth the fact that consumption is a contagious disease; that in persons in the last stages of the disease, death is hastened, rather than retarded, by coming to these high altitudes, and that destitute persons, unable to work, coming to this city with the disease, can not be cared for by the city, as such numbers come that it is entirely out of the question to do anything for them, and they will probably die on the streets if they do come. I held, and still hold, that the disease is very slightly, if at all, contagious here, as is evidenced by the fact that although from fifteen to thirty years is the age at which the disease is most frequently developed, not one of the thousands of white persons coming here in childhood and passing fifteen years, or coming here between fifteen and thirty, has developed the disease. The papers did not generally give a lucid account of the proceedings of the Board of Health. Amick saw the opportunity to work the press for a lot of free advertising and my impression is that his telegrams were "fakes." The most radical measure proposed by any member of the board was the adoption of the circular sent out last year by our State Health Officer, as expressing the views of the board. Quarantine against consumptives was never mentioned or thought of, but originated in the fertile brain of the Cincinnati quack, I suppose.

Yours truly,

W. M. YANDELL.

